# GABAergic Gene Expression in Postmortem Hippocampus from Alcoholics and Cocaine Addicts; Corresponding Findings in Alcohol-Naïve P and NP Rats

**DOI:** 10.1371/journal.pone.0029369

**Published:** 2012-01-13

**Authors:** Mary-Anne Enoch, Zhifeng Zhou, Mitsuru Kimura, Deborah C. Mash, Qiaoping Yuan, David Goldman

**Affiliations:** 1 National Institute on Alcohol Abuse and Alcoholism, National Institutes of Health, Bethesda, Maryland, United States of America; 2 School of Medicine, University of Miami, Miami, Florida, United States of America; Nathan Kline Institute and New York University School of Medicine, United States of America

## Abstract

**Background:**

By performing identical studies in humans and rats, we attempted to distinguish vulnerability factors for addiction from neurobiological effects of chronic drug exposure. We focused on the GABAergic system within the hippocampus, a brain region that is a constituent of the memory/conditioning neuronal circuitry of addiction that is considered to be important in drug reinforcement behaviors in animals and craving and relapse in humans.

**Methodology:**

Using RNA-Seq we quantified mRNA transcripts in postmortem total hippocampus from alcoholics, cocaine addicts and controls and also from alcohol-naïve, alcohol preferring (P) and non-preferring (NP) rats selectively bred for extremes of alcohol-seeking behavior that also show a general addictive tendency. A pathway-targeted analysis of 25 GABAergic genes encoding proteins implicated in GABA synthesis, metabolism, synaptic transmission and re-uptake was undertaken.

**Principal Findings:**

Directionally consistent and biologically plausible overlapping and specific changes were detected: 14/25 of the human genes and 12/25 of the rat genes showed nominally significant differences in gene expression (global p values: 9×10^−14^, 7×10^−11^ respectively). Principal FDR-corrected findings were that *GABBR1* was down-regulated in alcoholics, cocaine addicts and P rats with congruent findings in *NSF*, implicated in GABAB signaling efficacy, potentially resulting in increased synaptic GABA. *GABRG2*, encoding the gamma2 subunit required for postsynaptic clustering of GABAA receptors together with *GPHN*, encoding the associated scaffolding protein gephryin, were both down-regulated in alcoholics and cocaine addicts but were both up-regulated in P rats. There were also expression changes specific to cocaine addicts (*GAD1*, *GAD2*), alcoholics (*GABRA2*) and P rats (*ABAT*, *GABRG3*).

**Conclusions/Significance:**

Our study confirms the involvement of the GABAergic system in alcoholism but also reveals a hippocampal GABA input in cocaine addiction. Congruent findings in human addicts and P rats provide clues to predisposing factors for alcohol and drug addiction. Finally, the results of this study have therapeutic implications.

## Introduction

Chronic exposure to heavy alcohol and cocaine use is known to result in widespread neuronal adaptations. Some of these changes may be specific to alcohol or cocaine exposure but other changes may be more general, reflecting shared pathways in addiction. In addition, inter-individual neurobiological variation contributes to the heritability of addiction. We have previously published a global analysis of differential expression of 16,008 gene transcripts that were detected in the postmortem hippocampus of alcoholics, cocaine addicts and controls [1]. The aim of the current study was to distinguish changes in expression of gamma-aminobutyric acid (GABA) pathway genes that might be unique to alcoholics or cocaine addicts, and to identify changes common to both. Moreover, we attempted to distinguish trait and state effects for addiction in humans by identifying differences in GABAergic gene expression in alcohol naïve rats selectively bred for high (P) and low (NP) alcohol preference, a model for vulnerability to alcohol dependence. P rats have also shown evidence for addiction vulnerability to other substances including nicotine and cocaine [2].

There has been increasing interest in the role of the hippocampus in addiction, a disorder that involves changes in neuronal circuits involved in saliency/reward, motivation/drive, memory/conditioning and control/disinhibition [3], [4]. The hippocampus is implicated in long and short term episodic memory thereby playing a role in the processing of contextual cues within the memory/conditioning neuronal circuit that is considered to be important in drug reinforcement behaviors in animals and craving and relapse in humans [4]. Imaging studies in humans have shown that cue-elicited craving activates the hippocampus [3]. Addictive drugs impair neurogenesis in the adult hippocampus and there is increasing evidence to show that this may be associated with impairments of learning and memory [4]–[6]. Suppression of adult hippocampal neurogenesis has been shown to increase cocaine self-administration in rats [7]. Moreover, the hippocampus is part of the default mode network, a set of brain regions that exhibit resting state synchronized low frequency oscillations. The default mode network is impaired in addiction and it has been suggested that the altered resting state functional connectivity may underlie the heightened sensitivity to drug related cues and weakened cognitive control that is a hallmark of addiction [8]. Moreover, the hippocampus, together with the amygdala and the frontal cortex, sends projections to the nucleus accumbens that plays a critical role in the acute reinforcing effects of drugs [4], [9]. Hippocampal function is determined by the balance between excitatory pyramidal cell firing and their modulation by GABA-containing interneurons. Alcohol alters this balance and indeed the hippocampus has been implicated in the development of alcohol tolerance [10], [11]. GABAergic inhibition and hippocampal theta oscillations are both critical for synaptic plasticity and learning behaviors [12]. Cocaine depresses the GABA current of hippocampal neurons [13].

Rapid synaptic inhibition is mediated through GABAA receptors that are ligand-gated, chloride ion channels. GABAB receptors are G protein-coupled receptors that are present in almost all CNS neurons and regulate synaptic transmission and signal propagation. A substantial body of evidence from preclinical studies has implicated GABAA receptors in the acute and chronic effects of ethanol including tolerance, dependence and withdrawal [14]–[16]. Preclinical studies have implicated GABAB receptors in the rewarding effects of drugs of abuse [17], [18]. Indeed, GABAB agonists have been found to decrease alcohol consumption and craving in humans and severity of alcohol withdrawal symptoms in humans and rats [17], [19].

Preclinical studies have shown that changes in the expression of subunits of GABAA receptors are implicated in the development of ethanol tolerance and dependence as well as in the cortical excitability associated with withdrawal [14]. In this study we chose to do a comprehensive assessment of gene expression changes in GABAergic genes that encode proteins involved in GABA synthesis, transmission, transport and metabolism. We compared GABAergic gene expression in postmortem total hippocampus from eight alcoholics, eight cocaine addicts and eight controls using RNA-Seq, a deep-sequencing technology that maps the entire transcriptome and provides precise, accurate measurements of the level of transcripts [20], [21]. We used the same RNA Seq method to compare GABAergic gene expression in postmortem total hippocampus from eight selectively bred alcohol-preferring (P) and eight non-preferring (NP) rats.

## Methods

### Human hippocampal samples

Postmortem brain tissue was provided by the University of Miami Brain Bank. Since we used publicly available pathological specimens, our study was exempt from NIH Institutional Review Board (IRB) review. Research protocols at the University of Miami were approved by the University of Miami IRB. Brain tissues were removed from autopsy cases according to criteria described by the National Association of Medical Examiners Committee on Cocaine-related Deaths [22], [23]. Samples were limited to sudden death without medical intervention or prolonged agonal periods. The postmortem interval (PMI) was less than 24 hours. Brain pH was measured as a quality control for each sample with values >6.0. For further details see [24].

Regional samples of postmortem brain were taken from frozen coronal blocks based on surface and cytoarchitectural landmarks. The hippocampus was sampled bilaterally from coronal slices taken at the anterior level of the hippocampal body, including the dentate gyrus and the Cornu Ammonis fields CA1–CA4 and the subiculum.

The postmortem samples were taken from eight cocaine addicts, eight alcoholics and eight controls, all men. All subjects in the cocaine and alcohol groups met DSM-IV criteria for abuse or dependence. The cocaine addicts had long-standing histories of cocaine abuse and the deaths were attributed to cocaine intoxication. None of the cocaine addicts had a history of other drug misuse/dependence or of alcohol misuse/dependence and had not been drinking prior to death. The alcoholics had histories of chronic heavy alcohol consumption and all had enlarged livers: four had fatty livers, one had hepatic fibrosis. None had hepatic encephalopathy. None of the alcoholics had a history of drug misuse/dependence and a drug screen at the time of death was negative. The controls were age-matched and drug and alcohol free (negative urine screens, no history of licit or illicit drug use prior to death). Based on medical examiners' reports, next-of-kin informant reports, medical records and legal records, none of the subjects in this study had any other psychiatric disorders.

The mean (SD) ages were: cocaine addicts: 39.9 (4.9) yrs; alcoholics: 36.9 (9.5) years; controls: 37.5 (6.1) years. The mean (SD) PMIs were: cocaine addicts: 17.6 (3.0) hrs; alcoholics: 15.8 (3.5) hrs; controls: 17.1 (4.4) hrs. The ethnicity ratio: Caucasian or Caucasian/Hispanics:African Americans was as follows: cocaine addicts: 5∶3; alcoholics: 7∶1; controls: 5∶3.

### Hippocampal samples from P and NP rats

Hippocampal samples (total hippocampus) from selectively bred alcohol-preferring (P) and non-preferring (NP) rats, generation S70, were obtained from the Indiana University School of Medicine. The P and NP lines of rats were derived from a randomly bred, closed colony of Wistar rats by mass selection using a two-bottle free-choice paradigm with access to 10% (V/V) ethanol and water [25]. The sample consisted of eight alcohol preferring (P) and eight non-preferring (NP) male alcohol-naïve rats, all sacrificed at 90 days of age. For further details see [26].

### Construction of cDNA libraries

Details of the methods for construction of cDNA libraries have been described previously [1]. Briefly, total RNA was extracted from homogenized human postmortem hippocampal or dissected rat hippocampal tissue using guanidinium thiocyanate and phenol-based RNA extraction solution and purified on RNeasy spin columns. mRNA was isolated by oligo (dT)_25_ beads and fragmented. The fragmented mRNA was reverse transcribed and cDNA libraries were synthesized.

### High-throughput, massively parallel sequencing using an Illumina Genome Analyzer (GAIIx)

Sample preparation and sequencing on the Genome Analyzer (Illumina, San Diego, CA) were carried out according to the Illumina protocols with some modifications. Briefly, the double-stranded cDNA was treated with T4 DNA polymerase and the Klenow fragment for end repair. The 5′ end of the DNA fragments were then phosphorylated by T4 polynucleotide kinase, and an adenosine base was added to the 3′ end of the fragments by Klenow (3′-5′ exo^−^). A universal adaptor was then added to both ends of the DNA fragments by A-T ligation. Following 18 cycles of PCR with the Phusion DNA polymerase, the DNA library was then purified on a 2% agarose gel and fragments of 170–300 base-pair in size were recovered. Around 15 ng of the DNA library was then used for cluster generation on a grafted GAII Flow Cell, and sequenced on the Genome Analyzer for 36 cycles using the “Sequencing-by-synthesis” method.

### Sequence base-calling, mapping to genome, data normalization and statistical analysis

Sequences were called from image files with the Illumina Genome Analyzer Pipeline (GApipeline) and aligned to the reference genome (UCSC hg18 for human and UCSC rn4 for rat) using Extended Eland in the GApipeline. A total of 3 million uniquely mapped RNA-Seq reads for each human and rat sample were retrieved from export.txt files (output of Extended Eland). Based on their mapping locations, these selected reads were parsed with in-house Perl scripts to generate base coverage in WIG file format. After moving average smoothing, the chromosome locations of enrichment peaks were identified from pooled WIG files using in-house Perl scripts. The average sequencing reads of the most abundantly covered 50 bp in a single exon within an annotated Ref-Seq gene were counted for each sample. The read counts were then log2 transformed and normalized using quantile normalization (BioConductor limma package).

### Selection of candidate GABAergic genes

Table 1 lists the 25 GABAergic candidate genes that were selected for this study. The genes encode for GABA synthesis (*GLS*, *GAD1*, *GAD2*), metabolism (*ABAT*), vesicle transport (*SLC32A1*), and re-uptake at the synaptic cleft in neurons (*SLC6A1*) and glia (*SLC6A11*). *GABBR1* and *GABBR2* that encode the GABAB presynaptic and postsynaptic receptors were selected. Genes clustered on chromosomes 4, 5 and 15 that encode the GABAA postsynaptic receptors were included with the exception of *GABRA6* that is not expressed at detectable levels in the hippocampus. The trafficking of GABAA receptors is an important component in the regulation of plasticity of inhibitory synapses. The intracellular loop of GABAA receptor subunits provides protein-protein interactive domains involved in regulating receptor synaptic localization and intracellular trafficking For the purposes of this study, the genes encoding the key proteins that regulate synaptic localization were selected from the review by Chen and Olsen, 2007 [27]: *GPHN*, *NSF*, *UBQLN1*, *GABARAP*, *RDX* and *ZDHHC3* (Table 1).

**Table 1:** GABAergic Pathway Candidate Genes.

	GENES	PROTEINS
		**Presynaptic**
1	GLS	glutaminase
2	GAD1 (GAD67)	glutamic acid decarboxylase
3	GAD2 (GAD65)	glutamic acid decarboxylase
4	ABAT	4-aminobutyrate aminotransferase (GABA catabolism)
5	SLC32A1	VGAT: vesicular GABA transporter
6	SLC6A1	GAT1: plasma membrane GABA transporter (neurons)
7	SLC6A11	GAT3: plasma membrane GABA transporter (glia)
8	GABBR1	GABA_B_ receptor 1
9	GABBR2	GABA_B_ receptor 2
		**GABA_A_ receptor subunits**
10	GABRG1	Chr4: GABA_A_ γ1
11	GABRA2	Chr4: GABA_A_ α2
12	GABRA4	Chr4: GABA_A_ α4
13	GABRB1	Chr4: GABA_A_ β1
14	GABRB2	Chr5: GABA_A_ β2
15	GABRA1	Chr5: GABA_A_ α1
16	GABRG2	Chr5: GABA_A_ γ2
17	GABRB3	Chr15: GABA_A_ β3
18	GABRA5	Chr15: GABA_A_ α5
19	GABRG3	Chr15: GABA_A_ γ3
		**GABA_A_ receptor associated proteins**
20	GPHN	Gephryin
21	NSF	NSF: N-ethylmaleimide-sensitive fusion protein
22	UBQLN1	Plic-1: ubiquilin 1
23	GABARAP	GABARAP: GABA_A_ receptor associated protein
24	RDX	Radixin
25	ZDHHC3	GODZ: zinc finger, DHHC-type containing 3

GABRA6 (chr5) was not expressed at detectable levels in the hippocampal samples from humans or rats.

### Statistical analyses

Linear regression analyses were performed using JMP v7 with quantile normalized gene expression values as the dependent variable and diagnosis, PMI, age, and ethnicity (Caucasian/Hispanic or African American coded 1 or 2) as the independent variables. Age, ethnicity and PMI were included in the analyses if p≤0.1.

**Figure 1:**
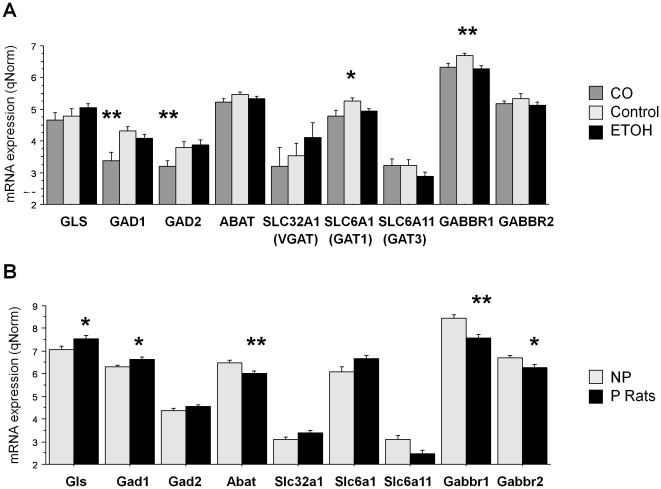
The Expression of Presynaptic/Synaptic GABAergic Genes in Human Samples and Rat Samples. ETOH = alcoholics; CO = cocaine addicts. Error bars: standard errors. ** FDR p<0.05; * FDR p≤0.06.

**Figure 2:**
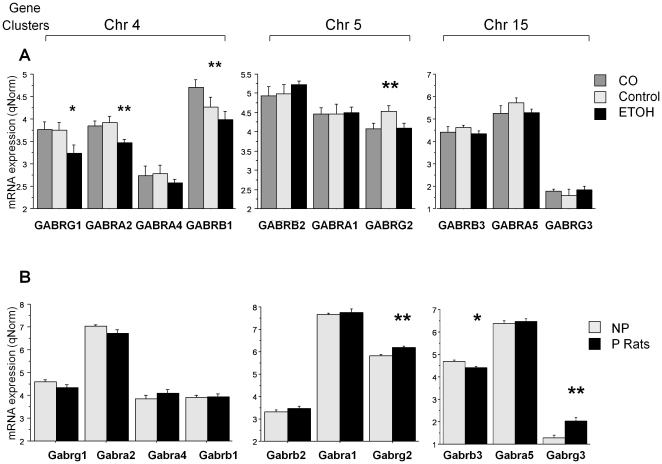
The Expression of GABAA Receptor Subunit Genes in Human Samples and Rat Samples. The genes are grouped in the chromosomal 4, 5 and 15 clusters. ETOH = alcoholics; CO = cocaine addicts. Error bars: standard errors. ** FDR p<0.05; * FDR p≤0.06.

**Figure 3:**
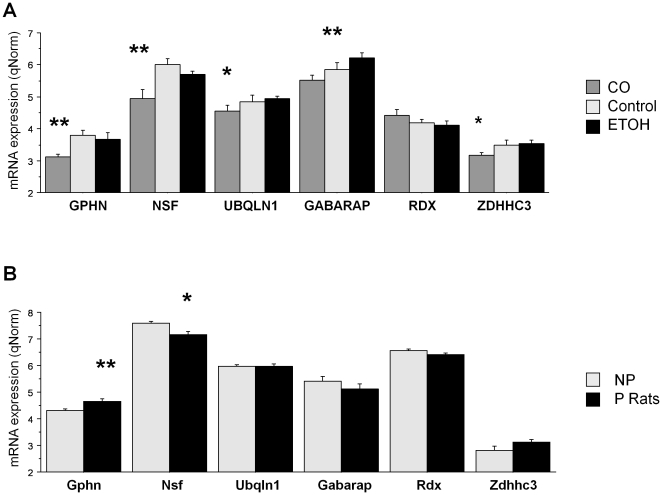
The Expression of Genes Encoding GABAA Receptor Associated Proteins in Human Samples and Rat Samples. ETOH = alcoholics; CO = cocaine addicts. Error bars: standard errors. ** FDR p<0.05; * FDR p≤0.06.

The aim of this exploratory study was to detect both overlapping and specific changes in gene expression in cocaine addicts and alcoholics. Therefore we based our analysis for each gene on the plots shown in Figures 1, 2 and 3. For example, in Figure 1 it can be seen that *GAD1* and *GAD2* have lower expression in cocaine addicts compared with both alcoholics and controls. As indicated in Table 2, the predominant analyses were: cocaine addicts vs. alcoholics+controls (7 analyses) (specific effect); cocaine addicts+alcoholics vs. controls (7 analyses) (overlapping effects); alcoholics vs. cocaine addicts+controls (4 analyses) (specific effect) and cocaine addicts vs. alcoholics (4 analyses) (specific effect). P values were corrected in the analyses in humans and in the analyses in P/NP rats using the False Discovery Rate (FDR) [28] based on 25 candidate genes. Global P values were calculated using the truncated product method [29], a modified Fisher's method (ftp://statgen.ncsu.edu/pub/zaykin/tpm). P values from all 25 independent tests with P<0.05 were combined and global significance was assessed by evaluating the distribution of their product.

**Table 2:** Analyses of Gene Expression Changes in Human and Rat Hippocampus.

	HUMANS				P vs NP RATS		
	Max Effect	F (df)	P value	FDR P	F(1,14)	P value	FDR P
**GLS**	CO vs ETOH	F(2,13) = 5.4	0.037	0.066	7.3	0.018	**0.050**
**GAD1**	CO vs CT+ETOH	F(1,22) = 12.9	0.002	**0.013**	7.2	0.018	**0.050**
**GAD2**	CO vs CT+ETOH	F(2,21) = 14.5	0.001	**0.013**	2.1	0.166	0.244
**ABAT**	CO vs CT	F(3,11) = 4.6	0.055	0.092	10.7	0.006	**0.030**
**SLC32A1**	CO vs ETOH	F(2,13) = 3.0	0.108	0.166	2.4	0.142	0.222
**SLC6A1**	CT vs CO+ETOH	F(1,22) = 6.3	0.020	**0.050**	4.7	0.047	0.098
**SLC6A11**	ETOH vs CT+CO	F(1,22) = 2.5	0.126	0.166	5.7	0.031	0.071
**GABBR1**	CT vs CO+ETOH	F(2,21) = 12.8	0.002	**0.013**	16.4	0.001	**0.025**
**GABBR2**	CT vs CO+ETOH	F(2,21) = 2.6	0.121	0.166	6.3	0.025	**0.063**
**GABRG1**	ETOH vs CT+CO	F(1,22) = 5.8	0.025	**0.056**	1.9	0.188	0.261
**GABRA2**	ETOH vs CT+CO	F(1,22) = 8.3	0.009	**0.028**	4.2	0.061	0.117
**GABRA4**	ETOH vs CT+CO	F(1,22) = 0.8	0.380	0.396	1.6	0.233	0.291
**GABRB1**	CO vs ETOH	F(1,14) = 7.5	0.016	**0.044**	0.0	0.900	0.900
**GABRB2**	ETOH vs CT+CO	F(1,22) = 1.3	0.270	0.300	1.6	0.220	0.290
**GABRA1**	CO vs CT vs ETOH	F(2,21) = 0.0	0.991	0.991	0.5	0.476	0.541
**GABRG2**	CT vs CO+ETOH	F(2,19) = 9.5	0.006	**0.021**	11.4	0.005	**0.030**
**GABRB3**	CT vs CO+ETOH	F(3,20) = 1.4	0.247	0.294	7.2	0.018	**0.050**
**GABRA5**	CT vs CO+ETOH	F(1,22) = 2.1	0.158	0.198	0.4	0.563	0.612
**GABRG3**	CT vs CO+ETOH	F(2,21) = 1.3	0.276	0.300	12.8	0.003	**0.025**
**GPHN**	CO vs CT+ETOH	F(1,22) = 10.5	0.004	**0.020**	15.2	0.002	**0.025**
**NSF**	CO vs CT+ETOH	F(2,21) = 23.6	<0.001	**<0.001**	8.0	0.014	**0.050**
**UBQLN1**	CO vs CT+ETOH	F(2,21) = 5.3	0.032	**0.062**	0.0	0.840	0.875
**GABARAP**	CO vs ETOH	F(1,14) = 10.8	0.005	**0.021**	1.1	0.319	0.380
**RDX**	CO vs CT+ETOH	F(1,22) = 2.5	0.125	0.166	2.6	0.129	0.215
**ZDHHC3**	CO vs CT+ETOH	F(1,22) = 5.7	0.027	**0.056**	3.3	0.091	0.163

Ethnicity, postmortem interval and age were included as covariates in the linear regression analyses if p≤0.1.

Both the uncorrected p values and the FDR corrected p values are shown. FDR corrected significant results and trend effects designated as p≤0.06 are shown in bold.

The analyses for ‘maximum effect’ in human samples were derived from the plots shown in Figures 1, 2 and 3.

ETOH = alcoholics; CO = cocaine addicts; CT = controls.

## Results

### Studies in Cocaine Addicts, Alcoholics and Controls

**Figure 4:**
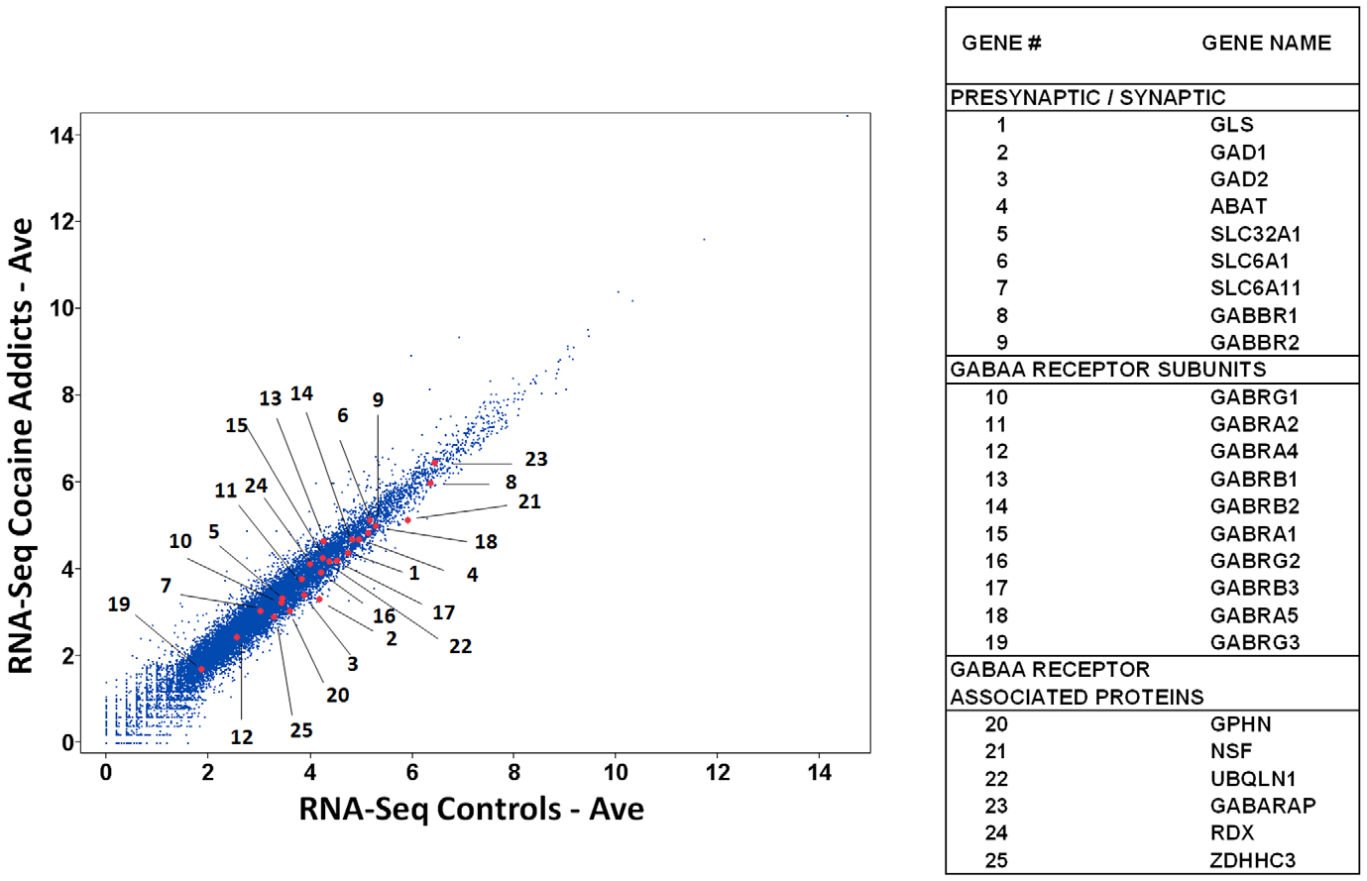
Expression of GABAergic Pathway Genes in the Human Hippocampus: Cocaine Addicts vs. Controls. The genome-wide expression levels of 16,008 transcripts, including the 25 GABAergic genes, are shown.

Expression of 16,008 gene transcripts was detected in the human hippocampus. The scatter plots of log2 transformed, quantile normalized expression levels of all these gene transcripts indicate that there is a high correlation between gene expression in cocaine addicts and controls (Figure 4) and between alcoholics and controls (Figure S1).

The global analyses on these 16,008 genes have been previously reported [1]. Gene expression changes detected by RNA-Seq were validated by quantitative RT-PCR for 11 transcripts in samples from eight controls and eight cocaine addicts. There was a strong correlation (R^2^ = 0.90) between the RNA-Seq and quantitative RT-PCR measurement indicating overall reliability of the RNA-Seq results [1].

Figures 4 and S1 indicate that, with the exception of *GABRG3*, the remaining 24 GABAergic pathway genes are moderately to highly expressed in the hippocampus. The highest expression values were detected for *GABBR1* and two genes, *NSF* and *GABARAP* that encode GABAA receptor associated proteins.

#### Differences in Expression of Gene Transcripts:

Full statistics, including FDR corrected p values, are presented in Table 2. Only FDR corrected p values are reported here.

#### Presynaptic/synaptic genes

From Figure 1A and Table 2 it can be seen that in cocaine addicts there was down-regulation of *GAD1* (p = 0.013) and *GAD2* (p = 0.013). These genes encode enzymes that synthesize GABA from glutamate. Both cocaine addicts and alcoholics had lower expression of *GABBR1* (p = 0.013) (Figures 1A, S2) and a trend (p = 0.050) towards lower expression of *SLC6A1* that encodes the GAT1 transporter that is responsible for re-uptake of GABA from the synaptic cleft.

#### GABAA receptor subunit genes

These genes are clustered on chromosomes 4, 5 and 15. From Table 2 and Figure 2A it can be seen that alcoholics showed down-regulation of *GABRA2* (p = 0.028) with a similar trend (p = 0.056) for the closely adjacent *GABRG1*. *GABRG2* was down-regulated in both alcoholics and cocaine addicts compared with controls (p = 0.021) (Figures 2A, S2). *GABRB1* was more highly expressed in cocaine addicts than in alcoholics (p = 0.044).

#### Genes encoding GABAA receptor associated proteins

From Table 2 and Figure 3A it can be seen that, relative to alcoholics and controls, cocaine addicts showed significant or trend down-regulation of *GPHN* (p = 0.02), *NSF* (p<0.001) (Figure S2), *ZDHHC3* (p = 0.056) and *UBQLN1* (p = 0.062). Alcoholics had increased expression of *GABARAP* relative to cocaine addicts (p = 0.021).

**Figure 5:**
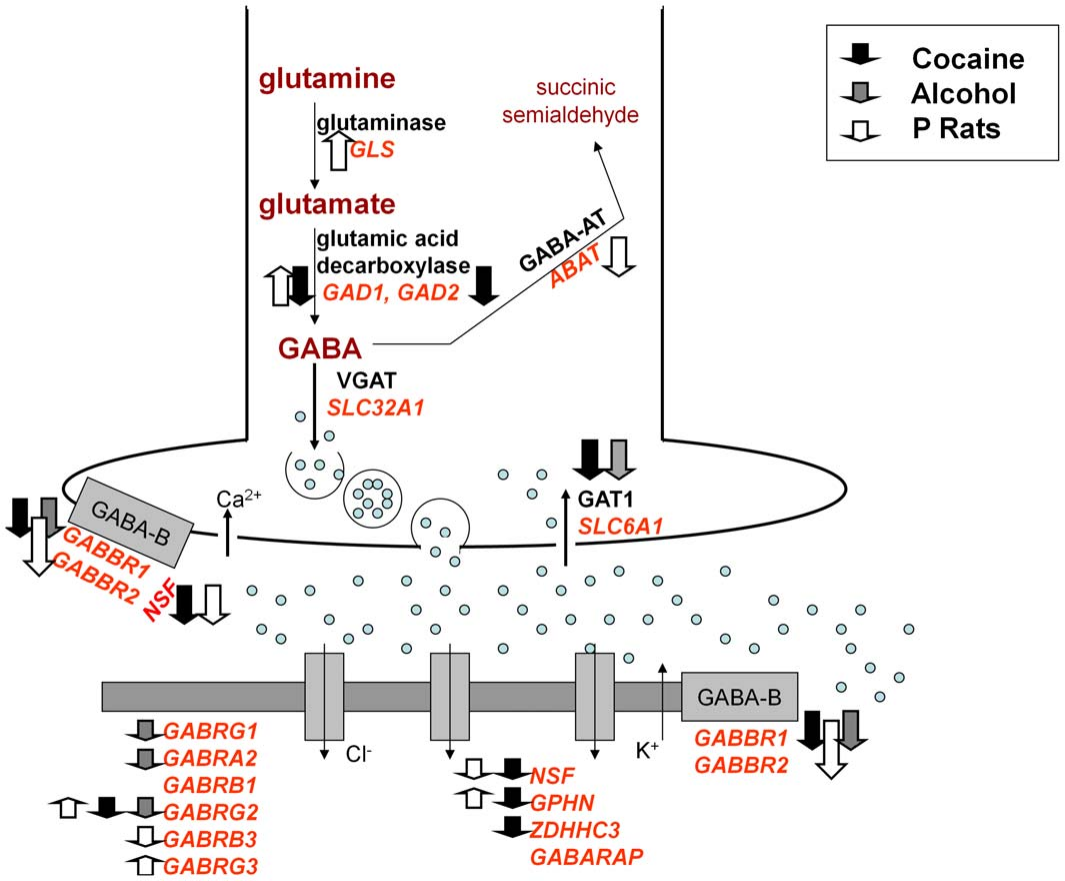
A Summary of Gene Expression Changes in Human Samples and in P Rats Relative to NP Rats. This schematic summarizes the gene expression changes shown in Table 2 (FDR p≤0.06).

A summary of the changes in gene expression are depicted in Figure 5. Figures S3 and S4 show the gene expression results for each individual cocaine addict, alcoholic and control.

### Studies in P and NP Rats

Expression of 11,406 gene transcripts was detected in the rat hippocampus. It should be noted that the read counts were independently normalized across the human samples and the rat samples and therefore gene expression levels can only be compared within each dataset and not across the two datasets.

#### Differences in Expression of Gene Transcripts:

Full statistics, including FDR corrected p values, are presented in Table 2. Only FDR corrected p values are reported here.

#### Presynaptic/synaptic genes

Table 2 and Figure 1B: In common with the alcoholics and cocaine addicts, P rats show significant down-regulation of *GABBR1* (p = 0.025) (Figure S2) compared with NP rats. In addition, they showed a trend effect for up-regulation of genes implicated in GABA synthesis: *GLS* (p = 0.050) and *GAD1* (p = 0.050) and significant down-regulation of *ABAT* (p = 0.030) that plays a role in GABA metabolism.

#### GABAA receptor subunit genes

Similar to the cocaine addicts and alcoholics, P rats show a significant difference in expression of *GABRG2* (p = 0.03). However this gene was up-regulated in P rats but down-regulated in alcoholics and cocaine addicts (Figures 2B and S2). P rats also show up-regulation of *GABRG3* (p = 0.025) together with a trend down-regulation of *GABRB3* (p = 0.050).

#### Genes encoding GABAA receptor associated proteins

In P rats, *GPHN* was up-regulated (p = 0.025) relative to NP rats, a finding that was congruent with the up-regulation of *GABRG2*. NSF was down-regulated (p = 0.050), a finding that was congruent with down-regulation of *GABBR1* (Figures 3B, S2).

A summary of the changes in gene expression in P rats relative to NP rats are depicted in Figure 5. Figures S5 and S6 show the gene expression results for each of the individual P and NP rats.

### Global P values

The selected GABAergic genes were strong candidates for involvement in addiction and in addictive tendencies. Indeed, 14/25 of the human genes and 12/25 of the rat genes showed nominally significant differences in gene expression. The global p value, calculated using the truncated product method [29] for overall differences in expression of the 25 genes in humans with alcohol or cocaine addiction compared with controls was p = 9.1×10^−14^. The global p value for overall differences in expression of the 25 genes in P rats compared with NP rats was p = 7.0×10^−11^.

## Discussion

The aim of this study of GABAergic gene expression in postmortem hippocampal samples was to distinguish changes that were common to both addictions as well as changes that might be unique to alcoholics or cocaine addicts. Many of these gene expression changes are likely to reflect tolerance to chronic, heavy use but it is possible that some differences between dependent individuals and controls may represent risk factors for addiction. Therefore we attempted to gain insight into trait versus state effects by comparing gene expression changes in alcoholics and cocaine addicts versus controls with gene expression changes in alcohol-naïve P and NP rats that were selectively bred for extremes of alcohol consumption behavior and are considered to be a realistic animal model of human alcohol dependence [25], [26], [30], [31]. P rats also show addiction vulnerability to other substances including cocaine [2].

A key finding of our study is that there was a robust down-regulation of *GABBR1* in both alcoholics and cocaine addicts compared with controls and in P rats compared with NP rats suggesting that *GABBR1* down-regulation could be a predictor for increased risk for addiction. A congruent finding is that *NSF*, which plays a role in the regulation of GABAB receptor signaling efficacy [32] was also down-regulated in both cocaine addicts and P rats.

GABAB receptors are ubiquitous G protein-coupled receptors that are heterodimers of GABAB1 and GABAB2 subunits. Presynaptic GABAB receptors repress Ca^2+^ influx, and therefore GABA release, by inhibiting the activity of voltage-gated calcium channels [33], [34]. In interpreting the results of our study it should be borne in mind that we only measured mRNA transcripts and not protein levels, however if decreased *GABBR1* expression results in down-regulation of GABAB receptors our identical findings in alcoholics, cocaine addicts and alcohol-naïve P rats suggest that this may be a marker for vulnerability to addiction. Indeed, accumulating evidence from studies in mice and rats (including Indiana P rats) shows that in the reverse situation of GABAB receptor up-regulation by agonists such as baclofen, there is a reduction of drug-related behaviors including alcohol consumption, relapse-like drinking and re-instatement of cocaine seeking behavior [17], [18], [35], [36], [37]. Moreover, baclofen decreases alcohol consumption, craving and severity of alcohol withdrawal symptoms in humans [17], [19]. By inference, down-regulation of GABAB receptors may increase the rewarding effects of drugs of abuse.

The second robust finding that was common to alcoholics, cocaine addicts and P rats was altered expression of *GABRG2* and also *GPHN* (encoding gephryin). *GABRG2* encodes the gamma2 subunit, a component of approximately 75% of all neuronal GABAA receptors that is essential for benzodiazepine sensitivity [38]. The gamma2 subunit is required for synaptic clustering of GABAA receptors and for the recruitment to postsynaptic sites of gephryin, a scaffolding protein that anchors GABAA receptors to the postsynaptic skeleton [39]–[43]. Both *GABRG2* and *GPHN* were significantly up-regulated in alcohol-naïve P rats relative to NP rats. In contrast, *GABRG2* was significantly down-regulated in alcoholics and cocaine addicts relative to controls and likewise *GPHN* was down-regulated in cocaine addicts. Under normal circumstances, only GABAA receptors that include a delta subunit in place of a gamma2 subunit are responsive to the usually low extrasynaptic GABA concentrations. However, it has been shown that increasing the ambient extracellular GABA concentration results in tonic activation of gamma2 subunit-containing GABAA extrasynaptic receptors [44], [45]. This could be one explanation for the up-regulation of *GABRG2* and *GPHN* in alcohol naïve P rats that, as mentioned earlier, may have chronically increased GABA levels in the synaptic cleft relative to NP rats. In contrast, the down-regulation of *GABRG2* (and *GPHN*) in alcoholics and cocaine addicts might be compensatory and a feature of tolerance to drug. This is supported by preclinical findings; for example cynomolgus macaque monkeys allowed to self-administer ethanol for 18 months show reduced gamma2 mRNA expression in the amygdala [46] and cultured rat hippocampal neurons exposed to ethanol for 5 days likewise showed reduced gamma2 mRNA levels [47].

In both alcoholics and cocaine addicts compared with controls there was a near significant (FDR p = 0.050) down-regulation of *SLC6A1* that encodes the principal neuronal GABA transporter (GAT1). Decreased levels of GAT1 could result in enhanced extracellular GABA levels, as has been shown to occur in GAT1 deficient mice [12]. Moreover, the effect of tiagabine, a GAT1 inhibitor is to reduce the subjective effects of cocaine in cocaine addicts [48]. This reduced response to drug or alcohol, i.e. ‘tolerance’, occurs with long-term use. Since there was no alteration in GAT1 expression in P rats, it is possible that the changes seen in humans may represent altered homeostasis seen in drug tolerance [49].

Changes specific to cocaine addiction were the down-regulation of *GAD1* and *GAD2*, genes that encode glutamic acid decarboxylase, the enzyme responsible for the majority of GABA synthesis from glutamate in the CNS. The fact that GAT1 deficient mice, although having enhanced extracellular GABA levels, show unaltered levels of GAD1 and VGAT [44], [50] suggests that this change in *GAD1* expression in cocaine addicts is likely to be independent and not compensatory for increased synaptic GABA. It is of interest that *GAD1* has also been shown to be down-regulated in the hippocampus of patients with schizophrenia and bipolar disorder [51].

Changes specific to alcoholism were the down-regulation of *GABRA2* (FDR p = 0.028) and a trend down-regulation of *GABRG1* (FDR p = 0.056). These closely adjacent genes in the chromosome 4 cluster respectively encode the GABAA receptor alpha2 and gamma1 subunits that have been robustly associated with alcohol use disorders and alcohol related phenotypes in human case-control studies (reviewed in [14]).

Compared with alcoholics, cocaine addicts showed significantly increased expression of *GABRB1* but significantly lower expression of *GABARAP* that encodes the protein GABARAP. Some [52] but not all [53] studies have shown that GABARAP anchors GABAA receptors to the postsynaptic cytoskeleton via high affinity binding to the gamma2 subunit and interactions with gephyrin and NSF. Certainly in cocaine addicts the *GABARAP* findings are congruent with those of *GPHN* and *NSF* but the relevance of the findings in alcoholics is not clear.

The Indiana P rats are considered to be a realistic animal model of human alcohol dependence because of the following features: (a) they consume 5–8 g of ethanol/kg/day, achieving a BAC of 50–200 mg% which is equivalent to human consumption of approximately 8–14 standard drinks/day; (b) they have been shown to consume ethanol for its CNS effects and not because of taste, odor or caloric properties and will work to obtain 10–40% V/V ethanol solutions despite free access to food and water; (c) they show increased stimulatory responses to low dose ethanol but lower response to the sedating/motor impairing effects; and (d) they show evidence of alcohol dependence characterized by: metabolic and functional tolerance, withdrawal (increased anxiety and lower seizure threshold) and relapse following prolonged abstinence [25], [26], [30], [31]. The P rat is more anxious than the NP rat, as assessed by three different measures, and responds to the anxiolytic effects of ethanol [54]. Therefore changes in the GABAergic system were expected. P and NP rats differ in their hippocampal theta currents [55] and this may be related in part to the differences in expression of *GABBR1* that we noted in our study [56]. Finally, compared with NP rats, P rats show increased nicotine self administration and relapse together with increased cocaine seeking during extinction and cocaine priming-induced reinstatement i.e. relapse vulnerability, indicating the likelihood of a general addictive tendency [2].

Some earlier studies have used microarrays to look at genome-wide differences in gene expression in the hippocampus of alcohol naïve P and NP rats [26]. A systems genetic analysis combining data from five strains of high and low alcohol consuming mice together with data from rats and humans identified the GABAergic transmission pathway as critical in the expression of the quantitative phenotype of alcohol consumption. The list of candidate genes included *ABAT*, *GAD1* and *GABRB2* [57], [58]. It is noteworthy that in our study *ABAT* and *GAD1* also differed in expression between P and NP rats.

One strength of our study is that the same RNA-Seq method was used in both human and rat postmortem samples and therefore comparisons of relative gene expression levels across humans and across rats were valid. RNA-Seq has a large dynamic range of expression levels over which transcripts can be detected unlike DNA microarrays that lack sensitivity for genes expressing at low or high levels [21]. One caveat for our study is that the results may be confined to the hippocampus since gene expression is likely to vary across brain regions [59]. Finally, the aim of this study was to detect both overlapping and specific changes in gene expression in cocaine addicts and alcoholics. Therefore we did not automatically perform expression analyses for each gene across all 3 groups (2df). Instead we based our analysis for each gene on the plots shown in Figures 1, 2 and 3 and as indicated in Table 2. It could be argued that our FDR correction should have been greater than for 25 analyses. Nevertheless, the fact that this exploratory approach yielded results in humans that were congruent with results in rats suggests that our significant findings are not likely to be false positives.

In conclusion, our study has shown that, at least within the hippocampus, chronic alcohol and cocaine exposure results in both overlapping and specific changes in expression of GABAergic genes. Similar findings in addiction-vulnerable rats provide clues as to predisposing factors for alcohol and drug addiction. The selected GABAergic genes were strong candidates for involvement in addiction and in addictive tendencies. Indeed, 14/25 of the human genes (global p = 9.1×10^−14^) and 12/25 of the rat genes (global p = 7.0×10^−11^) showed nominally significant changes in gene expression. Moreover, there was striking overlap in human and rat genes that showed no changes in expression; for example of the genes encoding the GABAA receptor subunits, changes were largely confined to genes encoding the gamma subunits and not the alpha or beta subunits (other than alpha2 in the alcoholics). Finally, it is remarkable that such strong and consistent findings were evident in the ‘snapshot in time’ captured in the human mRNA transcripts bearing in mind that other factors that might alter gene expression including lifetime stressors were unknown.

## Supporting Information

Figure S1 **Expression of GABAergic Pathway Genes in the Human Hippocampus: Alcoholics vs. Controls.** The genome-wide expression levels of 16,008 transcripts, including the 25 GABAergic genes, are shown.
(PDF)

Figure S2 **Congruent Findings in Gene Expression Changes in Humans and Rats.** CO = cocaine addicts (N = 8), CT = controls (N = 8), AD = alcoholics (N = 8). Alcohol-naïve rats: P = alcohol preferring (N = 8), NP = non-preferring (N = 8). qNorm = log2 transformed, quantile normalized mRNA expression levels.
(PDF)

Figure S3 **Gene Expression in Individual Cocaine Addicts Compared with Individual Controls.** CO1–CO9, CT1–CT9: 8 cocaine addicts and 8 controls respectively. qNorm = log2 transformed, quantile normalized mRNA expression levels.
(PDF)

Figure S4 **Gene Expression in Individual Alcoholics Compared with Individual Controls.** ETOH1–ETOH8, CT1–CT9: 8 alcoholics and 8 controls respectively. qNorm = log2 transformed, quantile normalized mRNA expression levels.
(PDF)

Figure S5 **Up-Regulated Genes in Individual P Rats Compared with Individual NP Rats.** NP1–NP 8, P1–P8: 8 NP rats and 8 P rats, respectively. qNorm = log2 transformed, quantile normalized mRNA expression levels.
(PDF)

Figure S6 **Down-Regulated Genes in Individual P Rats Compared with Individual NP Rats.** NP1–NP 8, P1–P8: 8 NP rats and 8 P rats, respectively. qNorm = log2 transformed, quantile normalized mRNA expression levels.
(PDF)
