# Supplementary material for: GABAergic Gene Expression in Postmortem Hippocampus from Alcoholics and Cocaine Addicts; Corresponding Findings in Alcohol-Naïve P and NP Rats
Source: PLoS One. 2012 Jan 13;7(1):e29369. doi: 10.1371/journal.pone.0029369 (PMC3258238; doi:10.1371/journal.pone.0029369)
Supplement: Figure S1 — Expression of GABAergic Pathway Genes in the Human Hippocampus: Alcoholics vs. Controls. The genome-wide expression levels of 16,008 transcripts, including the 25 GABAergic genes, are shown. (PDF) [file pone.0029369.s001.pdf]

FIGURE S1: Expression of GABAergic Pathway Genes in the Human Hippocampus: Alcoholics vs. Controls

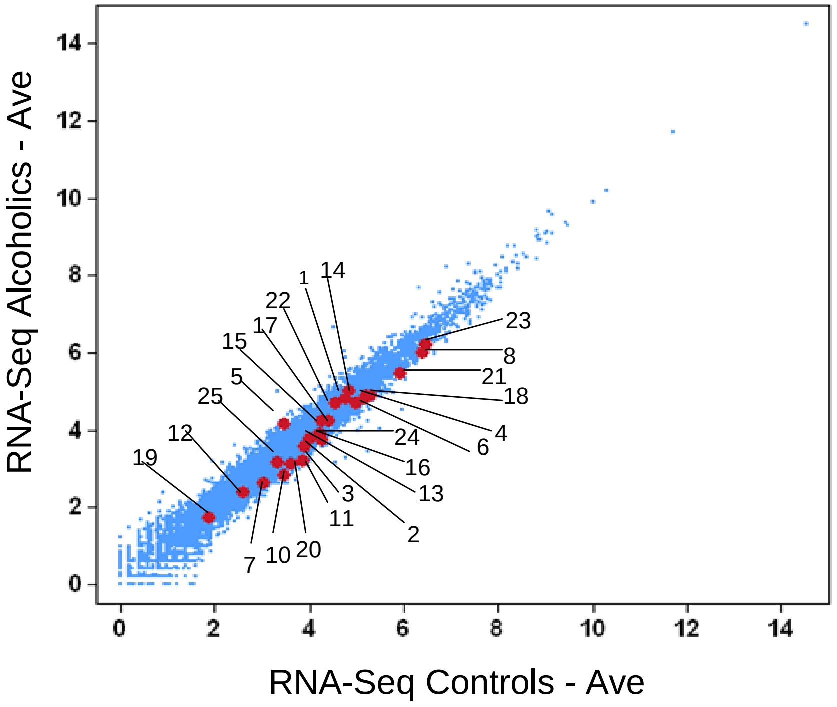

GENE #	GENE NAME
**PRESYNAPTIC / SYNAPTIC**	
1	GLS
2	GAD1
3	GAD2
4	ABAT
5	SLC32A1
6	SLC6A1
7	SLC6A11
8	GABBR1
9	GABBR2
**GABAA RECEPTOR SUBUNITS**	
10	GABRG1
11	GABRA2
12	GABRA4
13	GABRB1
14	GABRB2
15	GABRA1
16	GABRG2
17	GABRB3
18	GABRA5
19	GABRG3
**GABAA RECEPTOR ASSOCIATED PROTEINS**	
20	GPHN
21	NSF
22	UBQLN1
23	GABARAP
24	RDX
25	ZDHHC3

The genome-wide expression levels of 16,008 transcripts, including the 25 GABAergic genes, are shown
