# Supplementary material for: GABAergic Gene Expression in Postmortem Hippocampus from Alcoholics and Cocaine Addicts; Corresponding Findings in Alcohol-Naïve P and NP Rats
Source: PLoS One. 2012 Jan 13;7(1):e29369. doi: 10.1371/journal.pone.0029369 (PMC3258238; doi:10.1371/journal.pone.0029369)
Supplement: Figure S2 — Congruent Findings in Gene Expression Changes in Humans and Rats. CO = cocaine addicts (N = 8), CT = controls (N = 8), AD = alcoholics (N = 8). Alcohol-naïve rats: P = alcohol preferring (N = 8), NP = non-preferring (N = 8). qNorm = log2 transformed, quantile normalized mRNA expression levels. (PDF) [file pone.0029369.s002.pdf]

FIGURE S2: Congruent Findings in Gene Expression Changes in Humans and Rats

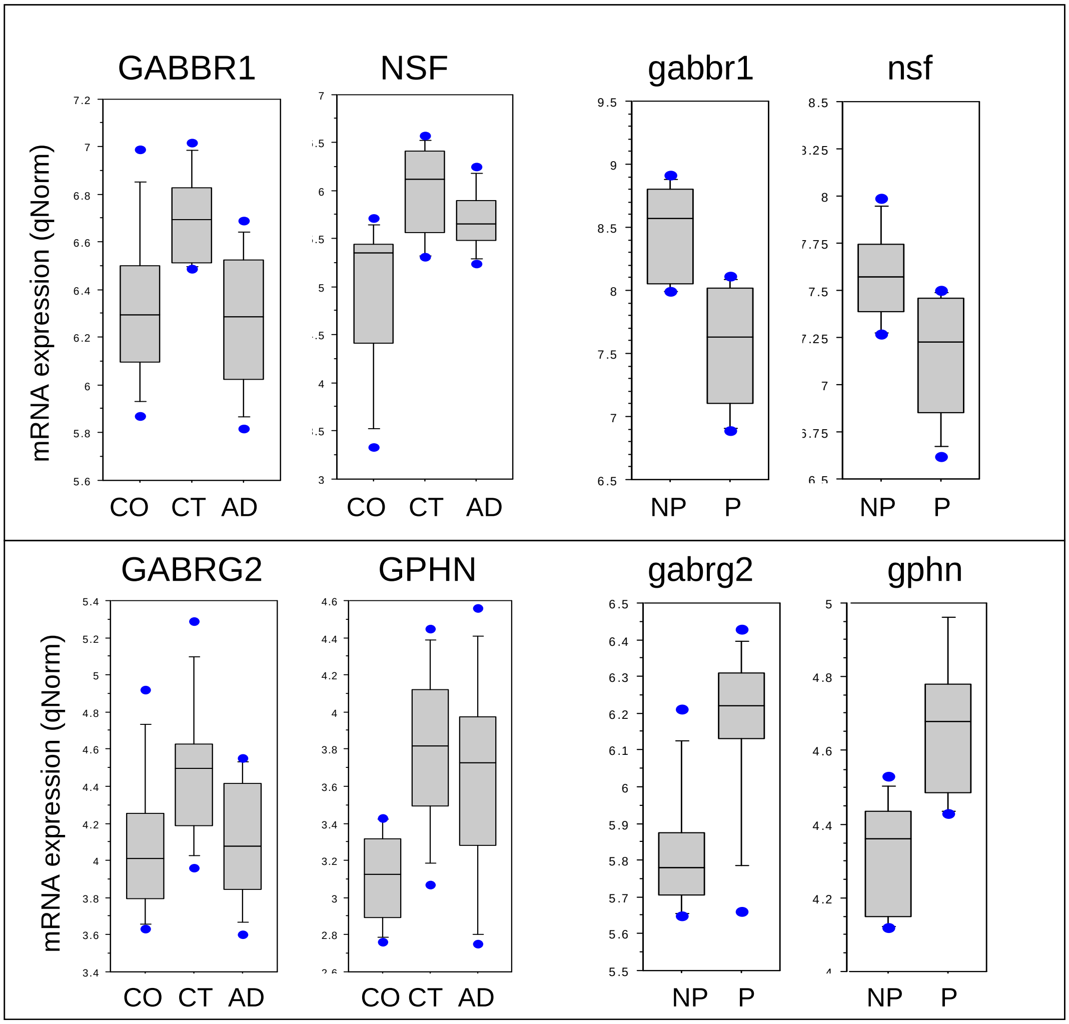

CO = cocaine addicts (N = 8), CT = controls (N = 8), AD = alcoholics (N = 8)
Alcohol-naïve rats: P = alcohol preferring (N = 8), NP = non-preferring (N = 8)
qNorm = log2 transformed, quantile normalized mRNA expression levels
