# Supplementary material for: GABAergic Gene Expression in Postmortem Hippocampus from Alcoholics and Cocaine Addicts; Corresponding Findings in Alcohol-Naïve P and NP Rats
Source: PLoS One. 2012 Jan 13;7(1):e29369. doi: 10.1371/journal.pone.0029369 (PMC3258238; doi:10.1371/journal.pone.0029369)
Supplement: Figure S4 — Gene Expression in Individual Alcoholics Compared with Individual Controls. ETOH1–ETOH8, CT1–CT9: 8 alcoholics and 8 controls respectively. qNorm = log2 transformed, quantile normalized mRNA expression levels. (PDF) [file pone.0029369.s004.pdf]

FIGURE S4: Gene Expression in Individual Alcoholics Compared with Individual Controls

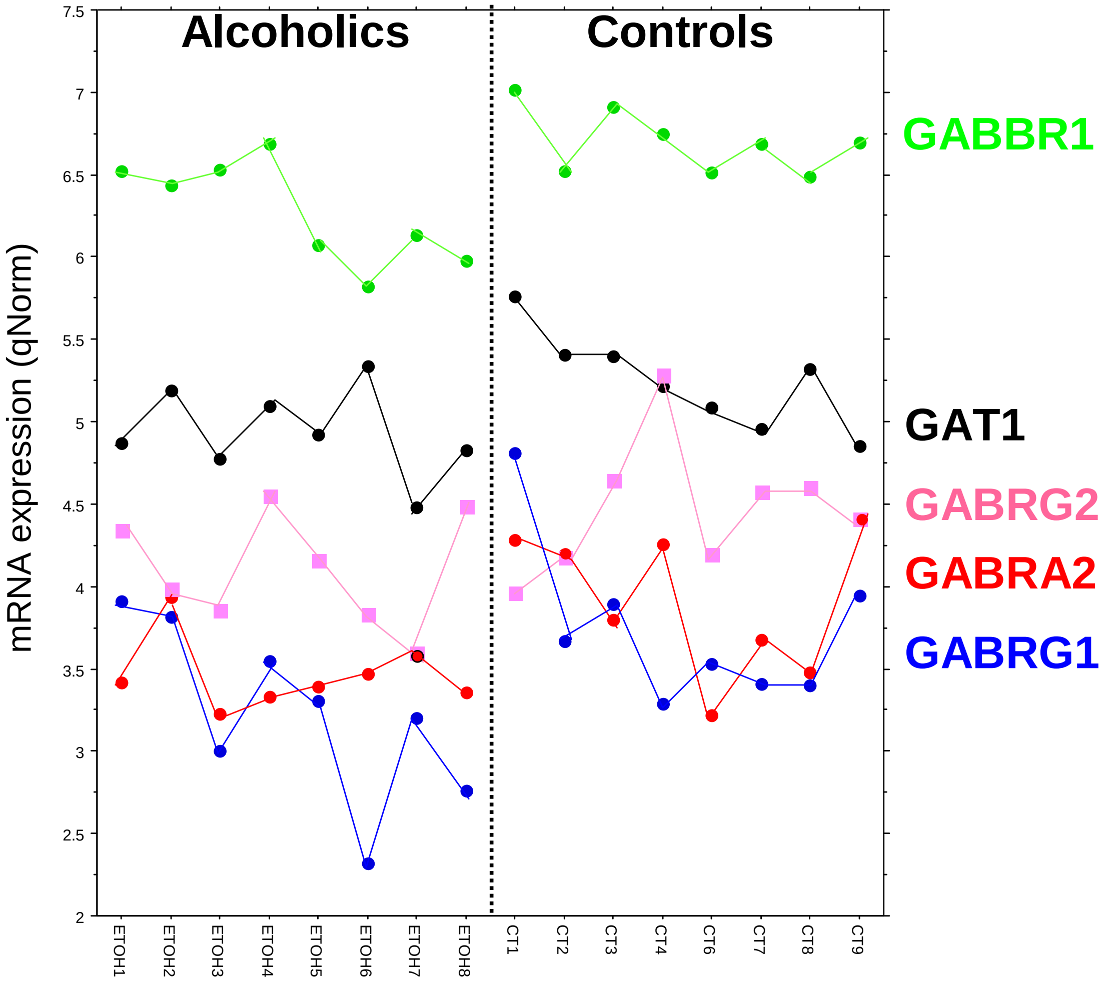

ETOH1 – ETOH8, CT1 – CT9: 8 alcoholics and 8 controls respectively
qNorm = log2 transformed, quantile normalized mRNA expression levels
