# Supplementary material for: GABAergic Gene Expression in Postmortem Hippocampus from Alcoholics and Cocaine Addicts; Corresponding Findings in Alcohol-Naïve P and NP Rats
Source: PLoS One. 2012 Jan 13;7(1):e29369. doi: 10.1371/journal.pone.0029369 (PMC3258238; doi:10.1371/journal.pone.0029369)
Supplement: Figure S6 — Down-Regulated Genes in Individual P Rats Compared with Individual NP Rats. NP1–NP 8, P1–P8: 8 NP rats and 8 P rats, respectively. qNorm = log2 transformed, quantile normalized mRNA expression levels. (PDF) [file pone.0029369.s006.pdf]

FIGURE S6: Down-Regulated Genes in Individual P Rats Compared with Individual NP Rats

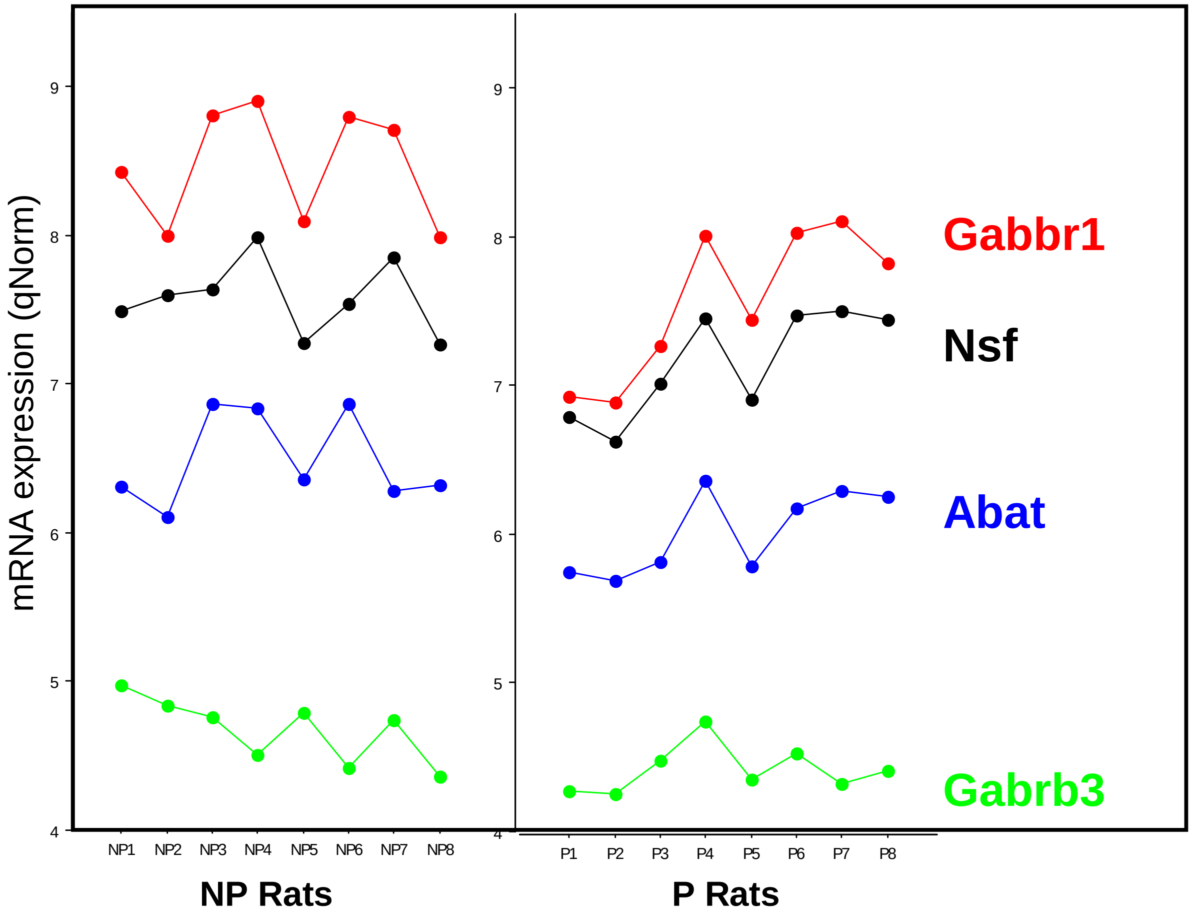

NP1 – NP 8, P1 – P8: 8 NP rats and 8 P rats, respectively
qNorm = log2 transformed, quantile normalized mRNA expression levels
